# Effects of pirfenidone on renal function in patients with interstitial pneumonia

**DOI:** 10.1080/0886022X.2021.1925297

**Published:** 2021-05-21

**Authors:** Jun Matsumoto, Keisuke Sunohara, Yoshiko Mori, Hiroshi Nagaya, Shinichiro Inaba

**Affiliations:** Department of Nephrology, Tosei General Hospital, Seto, Japan

To the Editor,

Declining renal function is associated with renal fibrosis, a common pathway of progressive renal diseases characterized by extracellular matrix accumulation. Transforming growth factor-β (TGF-β) increases the expression of individual matrix components [1]. Pirfenidone is a commonly used drug for treating interstitial pneumonia (IP) by inhibiting TGF-β production [2,3].

Interventional trials have been conducted on kidney diseases. In an open-label trial for focal segmental glomerulosclerosis (FSGS), the monthly change in estimated glomerular filtration rate (eGFR) improved from a median of −0.61 mL/min/1.73 m^2^ during the baseline period to −0.45, with pirfenidone therapy (*p* < .01) [4]. In a trial for diabetic nephropathy, the mean eGFR in the pirfenidone group increased by 3.3 over 12 months, while that in the placebo group decreased by 2.2 (*p* = .026) [5]. Despite these findings, pirfenidone had no effect on proteinuria in either trial.

Although these studies evaluated pirfenidone efficacy in patients with renal diseases characteristically associated with significant proteinuria, pirfenidone may inhibit the decline of renal function regardless of urinary protein levels. We retrospectively examined renal function changes before and after pirfenidone treatment in patients treated with pirfenidone for IP.

We reviewed 244 consecutive patients who were prescribed pirfenidone for IP at our hospital between 2009 and 2017. The inclusion criteria included an eGFR of <75 at the start of the treatment, while the exclusion criteria included discontinuation of pirfenidone medication, addition of angiotensin-converting enzyme inhibitors (ACE-I) or angiotensin II receptor blockers (ARB), hospitalization due to IP progression, and complications of any life-threatening disease during the survey period. This cohort included 93 patients after screening. The ethics committee of Tosei General Hospital approved the study (IRB #767).

All values were expressed as median (interquartile range) or number (%). The change in eGFR values during the 6 months before pirfenidone prescription, 6 months after the start of pirfenidone, and from 6 to 12 months after starting treatment were represented as ΔeGFR [−6 to 0], ΔeGFR [0 to 6], and ΔeGFR [6 to 12], respectively. We compared the ΔeGFR between the two sets using Friedman’s test, where *p*-values <.05 were considered statistically significant.

The clinical characteristics of the patients are shown in Table 1, which comprises two columns for all patients (eGFR <75, *n* = 93) and chronic kidney disease (CKD) patients (eGFR <60, *n* = 34). The median eGFR was 64.1 in all patients and 49.7 in CKD patients. Only a small proportion of the patients tested positive for proteinuria using the test strip method.

**Table 1.** Baseline characteristics of included patients.

	All patients (*N* = 93)	CKD patients (*N* = 34)
Patient characteristics*		
Age (years)	68 (62 − 73)	72 (66 − 75)
Male/Female	60/33	21/13
Body mass index (kg/m^2^)	24.0 (22.0 − 26.4)	24.1 (22.0 − 26.8)
Hypertension	28 (30%)	13 (38%)
Systolic blood pressure (mmHg)	137 (121 − 156)	144 (128 − 155)
Diastolic blood pressure (mmHg)	73 (67 − 81)	76 (68 − 81)
Diabetes mellitus	22 (24%)	14 (41%)
Hyperlipidemia	23 (25%)	11 (32%)
Cardiovascular diseases	10 (11%)	7 (21%)
Use of ACE-I or ARB	28 (30%)	16 (47%)
Immunosuppressive therapy	26 (28%)	12 (35%)
Pirfenidone doses (mg)	1540	1435
Serological tests		
Creatinine (mg/dL)	0.88 (0.75 − 0.97)	1.03 (0.87 − 1.22)
eGFR (mL/min/1.73m^2^)	64.1 (54.8 − 68.3)	49.7 (45.4 − 57.0)
Blood urea nitrogen (mg/dL)	15.1 (12.4 − 18.0)	18.1 (15.7 − 24.1)
Albumin (g/dL)	4.1 (3.9 − 4.3)	4.2 (3.9 − 4.3)
Hemoglobin (g/dL)	13.6 (12.9 − 14.4)	13.5 (12.4 − 14.3)
Glucose (mg/dL)	109 (97 − 132)	109 (96 − 131)
PaO2 (Torr)	78.3 (73.1 − 83.7)	78.9 (74.0 − 90.5)
**Urinalysis**		
Occult blood	10 (11%)	5 (15%)
Proteinuria	6 (6%)	3 (9%)

*Data are presented as median (interquartile range) or number (%).

ACE-I: angiotensin-converting enzyme inhibitor; ARB: angiotensin II receptor blocker.

**Figure 1.**
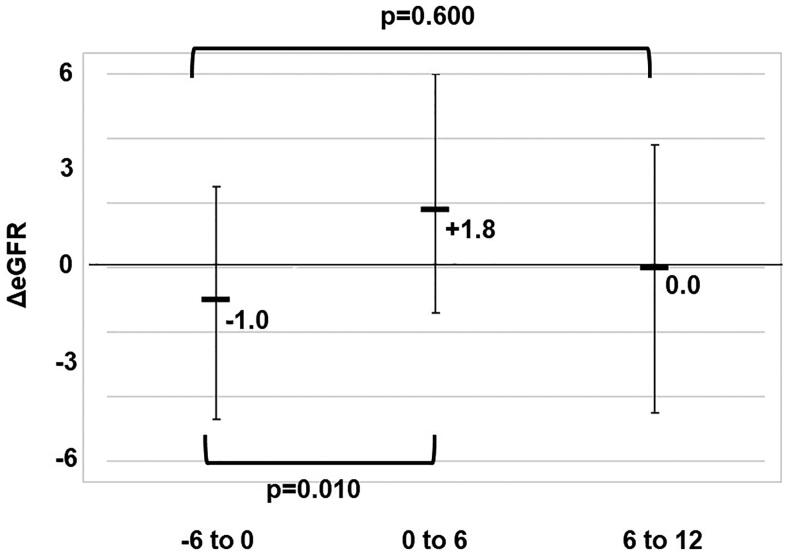
Comparison of ΔeGFR [0 to 6] and ΔeGFR [6 to 12] with ΔeGFR [−6 to 0] in patients with eGFR <75 mL/min/1.73m^2^.The values of ΔeGFR [−6 to 0], ΔeGFR [0 to 6], and ΔeGFR [6 to 12] were −1.0, +1.8, and 0.0, respectively. Pirfenidone significantly suppressed renal function decline for up to 6 months after starting treatment (*p* = .010).

We compared ΔeGFR [0 to 6] and ΔeGFR [6 to 12] with ΔeGFR [−6 to 0] in all patients and CKD patients. As shown in Figure 1, the eGFR values 6 months before treatment and at 0, 6, and 12 months after the start of treatment in all patients were 62.2, 64.1, 63.7, and 63.1, respectively. The ΔeGFR [−6 to 0], ΔeGFR [0 to 6], and ΔeGFR [6 to 12] values were −1.0, +1.8, and 0.0, respectively. Pirfenidone significantly suppressed renal function decline for up to 6 months after starting treatment (*p* = .010).

**Figure 2.**
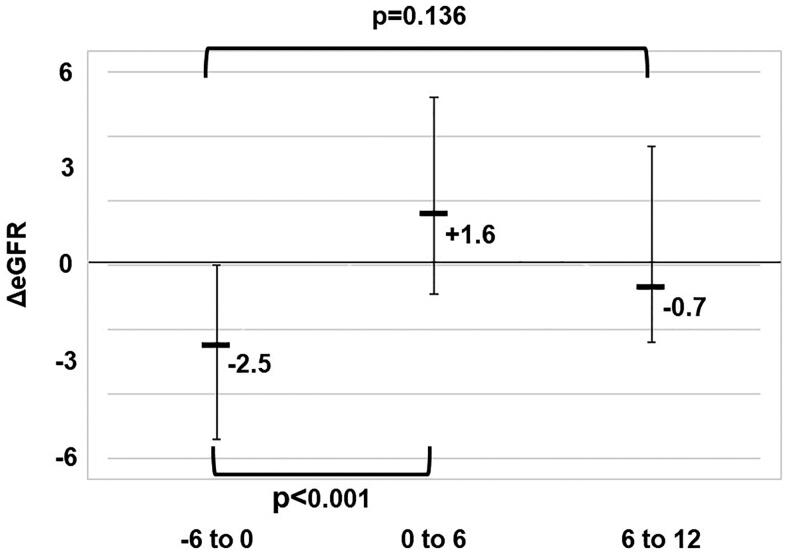
Comparison of ΔeGFR [0 to 6] and ΔeGFR [6 to 12] with ΔeGFR [−6 to 0] in patients with eGFR <60 mL/min/1.73m^2^.The values of ΔeGFR [−6 to 0], ΔeGFR [0 to 6], and ΔeGFR [6 to 12] were −2.5, +1.6, and −0.7, respectively. Compared to baseline values, pirfenidone significantly suppressed the renal function decline up to 6 months after starting treatment (*p* < .001), additionally showing tendency to suppress renal function even at 12 months after treatment (*p* = .136).

On the other hand, the eGFR values 6 months before treatment at 0, 6, and 12 months after the start of treatment in CKD patients were 53.9, 49.7, 53.1, and 51.8, respectively. The ΔeGFR [−6 to 0], ΔeGFR [0 to 6], and ΔeGFR [6 to 12] values were −2.5, +1.6, and −0.7, respectively (Figure 2). Compared to baseline values, pirfenidone significantly suppressed renal function decline up to 6 months after starting treatment (*p* < .001), additionally showing tendency to suppress renal function even at 12 months after treatment (*p* = .136).

A new therapeutic agent that can suppress renal decline in patients with CKD is urgently needed as the number of patients with end-stage renal failure continues to increase. Consistent with previous clinical investigations, the current study revealed that pirfenidone suppresses renal decline in patients with lower eGFR with little or no proteinuria.

Moreover, several animal studies have demonstrated the effectiveness of pirfenidone in various renal diseases. In 5/6 nephrectomy rats, pirfenidone suppressed collagen accumulation in the remaining kidney [6]. Additionally, pirfenidone improved tubulointerstitial fibrosis in rats with chronic cyclosporine nephrotoxicity [3], and improved intratubular fibrosis in rats with unilateral ureteral obstruction [7]. These favorable effects of pirfenidone in rats may support our findings that the deterioration of renal function was suppressed in patients without significant proteinuria.

In glomerular lesions, pirfenidone may also exhibit favorable effects since pirfenidone in db/db mice, a type 2 diabetes model, was found to inhibit mesangial substrate growth by suppressing TGF-β production [8]. In rats with anti-glomerular basement membrane nephritis, the prophylactic administration of pirfenidone significantly suppressed the progression of proteinuria; however, when it was administered after nephritis onset, proteinuria did not improve, although fibrosis was suppressed [9]. Pirfenidone might ameliorate glomerular fibrosis but may not show a definite ability to reduce proteinuria. Given that preventive pirfenidone administration may protect against proteinuria, pirfenidone pretreatment might be useful for preventing proteinuria in secondary glomerular diseases such as diabetic nephropathy.

Our study revealed that pirfenidone suppressed the decline in renal function for 6 months after starting treatment in patients with a lower eGFR. In CKD patients with eGFR <60, renal decline was suppressed for 6 months or longer after starting treatment. Pirfenidone was more effective in protecting against renal decline in CKD patients, indicating that it may be effective in patients with advanced fibrosis. Future studies with a larger number of cases may reveal significant differences after long-term treatment.
